# Improving Vehicle Heading Angle Accuracy Based on Dual-Antenna GNSS/INS/Barometer Integration Using Adaptive Kalman Filter

**DOI:** 10.3390/s24031034

**Published:** 2024-02-05

**Authors:** Hongyuan Jiao, Xiangbo Xu, Shao Chen, Ningyan Guo, Zhibin Yu

**Affiliations:** 1 School of Technology, Beijing Forestry University, Beijing 100083, China; jiaohongyuan@bjfu.edu.cn (H.J.); chenshao@bjfu.edu.cn (S.C.); 2 School of Information and Communication Engineering, Beijing University of Posts and Telecommunications, Beijing 100876, China; 3 Institute of Space Science and Applied Technology, Harbin Institute of Technology, Shenzhen 518055, China; yuzb@hit.edu.cn

**Keywords:** vehicle heading, dual-antenna GNSS/INS/barometer integration, adaptive Kalman filter, kinematic constraint, random sample consensus

## Abstract

High-accuracy heading angle is significant for estimating autonomous vehicle attitude. By integrating GNSS (Global Navigation Satellite System) dual antennas, INS (Inertial Navigation System), and a barometer, a GNSS/INS/Barometer fusion method is proposed to improve vehicle heading angle accuracy. An adaptive Kalman filter (AKF) is designed to fuse the INS error and the GNSS measurement. A random sample consensus (RANSAC) method is proposed to improve the initial heading angle accuracy applied to the INS update. The GNSS heading angle obtained by a dual-antenna orientation algorithm is additionally augmented to the measurement variable. Furthermore, the kinematic constraint of zero velocity in the lateral and vertical directions of vehicle movement is used to enhance the accuracy of the measurement model. The heading errors in the open and occluded environment are 0.5418° (RMS) and 0.636° (RMS), which represent reductions of 37.62% and 47.37% compared to the extended Kalman filter (EKF) method, respectively. The experimental results demonstrate that the proposed method effectively improves the vehicle heading angle accuracy.

## 1. Introduction

With the advancement of vehicle intelligence, real-time attitude based on available measurements plays a vital role in reliable path planning, decision making, and controls in autonomous vehicles [1]. Considering the actual conditions of the vehicle in motion, the estimation of vehicle attitude is mainly determined by the accuracy of the heading angle [2]. A variety of sensors can be utilized to measure the heading angle, such as GNSS (Global Navigation Satellite System), INS (Inertial Navigation System), magnetometers, vision sensors, laser radar, wireless signals, and so on [3,4,5,6,7,8,9,10,11]. In the above-mentioned methods, the integration of GNSS/INS is one of the most common approaches in the field of automotives [12].

However, some essential state variables, such as heading (error), are not well observed in some specific driving scenarios while fusing GNSS and INS data [13]. For a low-cost inertial measurement unit (IMU), this phenomenon is even more pronounced [14]. Therefore, in order to improve the accuracy of the heading angle and increase the robustness of the system, it is necessary to add other sensors and adopt innovative algorithms.

GNSS velocity measurements can be accurate because a GNSS receiver determines velocity based on the Doppler shift of GNSS carrier waves or by differencing consecutive GNSS carrier wave measurements [15]. Therefore, two GNSS receivers were utilized to measure the velocity of a vehicle and calculate the vehicle heading angle based on kinematic relationships [16]. At the same time, increasing the number of GNSS receivers results in an increase in costs. As technology evolves, dual-antenna GNSS receivers, utilizing baseline orientation to determine heading angles, are widely applied for integration with an IMU [17,18]. A wheel speed sensor (WSS) is another option to replace the second GNSS receiver because it can measure the speed of individual wheels. But velocities from WSSs need to be converted to the local navigation frame (*n*-frame) with the aid of an IMU [19]. It is also practical to use a magnetometer as an additional sensor to measure the heading angle. To reject the magnetic disturbances from other components, a new stochastic filter was designed and integrated into a Kalman filter framework [20]. To suppress the divergence of the heading angle, an effective approach is the kinematic model-based method, which is a simple vehicle model that correlates the vehicle’s longitudinal and lateral velocities with longitudinal and lateral accelerations and the heading rate [21]. A steering constraint based on a kinematic model was designed to enhance the heading angle estimation accuracy of land vehicles [22]. However, this method is only applicable to stationary or low-speed states. Inspired by vehicle kinematics, a novel method of heading angle estimation based on Zero-Heading angle-Variation-Constraint and Sequential-Adaptive Unscented Kalman Filter algorithms is proposed for solving the problem of heading angle unobservability and the instability of the filtering [23]. Owing to the presence of heading angular outputs from dual-antenna GNSS and INS systems, the mounting angle errors will reduce the heading angle accuracy [24]. GNSS antennas usually have a regular shape, and the mounting angle can be measured using optical methods. But this method requires specialized equipment to be prepared in advance. Heading angle misalignment between a dual-antenna GNSS and a vehicle frame was also estimated using a robust regression method [13]. The influence of random noise and outliers was effectively suppressed through this algorithm in the heading angle measurement. For mounting angle errors between the IMU frame and the vehicle frame, the dead reckoning method in a straightforward Kalman filter was a conventional and proven method. But formal experiments require a period of time for filter convergence [25,26]. A novel model was proposed to associate the INS heading angle error with the position and velocity by a lever arm [27]. However, experimental validation was set up only for large angular errors. To address smaller error angles, an error-state Kalman filter using vehicle velocity and non-holonomic constraints was developed to estimate IMU mounting angles [28].

Inspired by [18], a dual-antenna GNSS receiver is added to this study to improve the vehicle heading angle accuracy and supply the initial heading. The barometer that calculates elevation changes by obtaining the difference in air pressure values at different locations is one of the common sensors in consumer-grade devices. However, integrated GNSS/INS/Barometer navigation in vehicles has been under-explored. Based upon the foregoing, the main contributions of this study are summarized as follows:

1. A random sample consensus (RANSAC) method is applied to improve the initial heading angle accuracy and to ensure the initial attitude accuracy of the INS.
2. GNSS heading angle obtained by a dual-antenna orientation algorithm is added as a measurement variable in the integrated navigation system based on an adaptive Kalman filter (AKF) in addition to position and velocity. Furthermore, velocity and position errors caused by the lever arm are corrected. The kinematic constraint of zero velocity in the lateral and vertical directions of vehicle movement is used to enhance the accuracy of the measurement model.
3. A barometer is added to the traditional GNSS/INS integrated navigation system to enhance the reliability of the system. When none of the GNSS receiver data are available, the barometer and INS data are used to ensure a short-time accuracy.

The remainder of this paper is organized as follows: The Section 2 describes the basic concepts, the dual-antenna orientation algorithm, and the fusion process using AKF. The Section 3 introduces the experimental platform and analyzes the error of the experimental results in the open and occluded environment. The Section 4 summarizes this work.

## 2. Methodology Design

**Figure 1:**
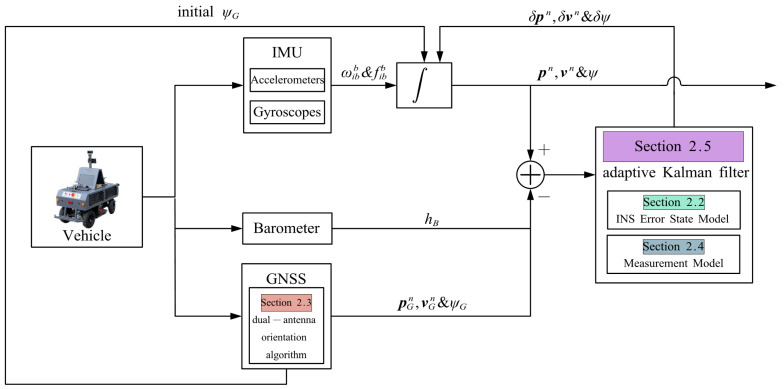
The architecture of the dual-antenna GNSS/INS/Barometer integrated navigation system. ${*}_{G}$ represents values measured by GNSS. δ represents the error value.

The architecture of the GNSS/INS/Barometer integration is illustrated in Figure 1. $\omega_{ib}^{b}$ represents the angular velocity of the IMU body with respect to inertial space in IMU body axes measured by the gyroscopes. $f_{ib}^{b}$ represents the specific force of the IMU body with respect to inertial space in the IMU. $v^{n}$ and $p^{n}$ are the three-dimensional velocity and position. ψ and h represent the heading angle and elevation.

To ensure the accuracy of the vehicle heading angle output by the proposed algorithm, the heading angle mounting error is corrected by an error-state Kalman filter using kinematic constraints. Due to the rectangular body of the intelligent automobile and the presence of the antenna-limiting holes, the calibration of the GNSS antenna can be performed with millimeter accuracy using a scale at the time of installation.

### 2.1. Reference Coordinate System for Integrated Navigation System

**Figure 2:**
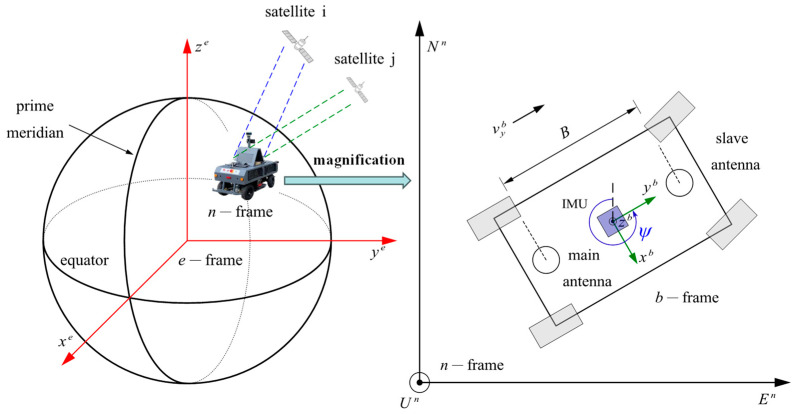
Diagram of the coordinate systems.

Integrated navigation requires a set of unified coordinate systems to accurately represent the state of the vehicle. The common coordinate systems are Earth-Centered Earth-Fixed (*e*-frame), local navigation frame (*n*-frame), and body frame (*b*-frame). In the *n*-frame, the *x*-axis, *y*-axis, and *z*-axis point east, north, and up, respectively. The *b*-frame is the right-handed 3D Cartesian frame rigidly connected to the vehicle with the *x*-axis, *y*-axis, and *z*-axis in the vehicle’s right, front, and up directions, respectively. The diagram of the coordinate system is shown in Figure 2.

To establish the connection between the *n*-frame and *b*-frame, three Euler angles (roll, pitch, and heading) are used to construct the coordinate transformation matrix. In the *n*-frame, the matrix $C_{n}^{b}$ is obtained by rotating the *z*-*x*-*y* axes (heading–pitch–roll) sequentially. The coordinate transformation matrix $C_{n}^{b}$ is expressed as

(1) $$C_{n}^{b}=\left[\begin{array}{ccc} \cos\phi \cos\psi -\sin\gamma \sin\theta \sin\psi & \cos\phi \sin\psi +\sin\gamma \sin\theta \cos\psi & -\sin\phi \cos\theta \\ -\cos\theta \sin\psi & \cos\theta \cos\psi & \sin\theta \\ \sin\phi \cos\psi +\cos\gamma \sin\theta \sin\psi & \sin\phi \sin\psi -\cos\phi \sin\theta \cos\psi & \cos\phi \cos\theta \end{array}\right]$$

where ϕ, θ, and ψ are the roll, pitch, and heading angles, respectively.

### 2.2. INS Error-State Model Development

#### 2.2.1. RANSAC Algorithm

For consumer-grade IMUs, initial alignment does not obtain a heading angle. Therefore, an accurate initial heading angle from the GNSS is critical for attitude updates.

The initial heading angle is modeled as

(2) $$\psi_{initial\_G}=\psi_{initial}+\chi$$

where $\psi_{initial\_G}$ is measured by GNSS, $\psi_{initial}$ is the true value, and χ represents measurement noise.

A RANSAC method is applied to estimate $\psi_{initial}$. To tackle the abnormal values in GNSS measurements, all data are divided into inliers and outliers based on a threshold ε [29]:

(3) $$\psi_{initial\_G}=\{\begin{array}{c} \mathrm{inliers}\begin{array}{ccc} & d\leq \varepsilon & \end{array}\\ outliers\begin{array}{ccc} & d>\varepsilon & \end{array} \end{array}$$

where d means the distance from the point to the line formed by the chosen two points.

After iteration, the model containing the most inliers is chosen as the true value model.

#### 2.2.2. INS Error-State Model

According to the mechanical choreography of inertial navigation, the non-linear error model is linearized. The error model can be expressed as [30]

(4) $$\dot{\alpha }=-\omega_{in}^{n}\times \alpha +\delta \omega_{in}^{n}-C_{b}^{n}\delta \omega_{ib}^{b}$$

(5) $$\delta {\dot{v}}^{n}=f^{n}\times \alpha -\left(2\delta \omega_{ie}^{n}+\delta \omega_{en}^{n}\right)\times v^{n}-\left(2\omega_{ie}^{n}+\omega_{en}^{n}\right)\times \delta v^{n}+\delta f^{n}+\delta g^{n}$$

(6) $$\delta {\dot{p}}^{n}=M_{pv}\delta v^{n}+M_{pp}\delta p^{n}$$

where α is the misalignment angle and $\alpha ={\left[\begin{array}{ccc} \delta \phi & \delta \theta & \delta \psi \end{array}\right]}^{T}$, $\delta v^{n}$ is the velocity error in the *n*-frame, $\delta v^{n}={\left[\begin{array}{ccc} \delta v_{E}^{n} & \delta v_{N}^{n} & \delta v_{U}^{n} \end{array}\right]}^{T}$, $\delta p^{n}$ is the position error, and $\delta p^{n}={\left[\begin{array}{ccc} \delta L & \delta \lambda & \delta h \end{array}\right]}^{T}$. $M_{pv}$ (1,2) = $\frac{1}{R_{M}+h}$, $M_{pv}$ (2,1) = $\frac{\sec L}{\left(R_{N}+h\right)}$, $M_{pv}$ (3,3) = 1, $M_{pp}$ (1,3) = $-\frac{V_{N}}{{\left(R_{M}+h\right)}^{2}}$, $M_{pp}$ (2,1) = $\frac{V_{E}\sec L\tan L}{\left(R_{N}+h\right)}$, $M_{pp}$ (2,3) = $-\frac{V_{E}\sec L}{{\left(R_{N}+h\right)}^{2}}$, and the other elements in the $M_{{pv}_{3\times 3}}$ and $M_{{pp}_{3\times 3}}$ are zero.

Generally, the gyro constant drift $\varepsilon^{b}$ and accelerometer constant bias $\nabla^{b}$ are described by random constants as follows [12]:

(7) $$\delta {\dot{\varepsilon }}_{i}^{b}=0\;(i=x,y,z)$$

(8) $$\delta {\dot{\nabla }}_{i}^{b}=0\;(i=x,y,z)$$

The system-state vector of the INS/GNSS integrated system is defined as

(9) $$x(t)={\left[\begin{array}{lllllllll} \alpha_{E} & \alpha_{N} & \alpha_{U} & \delta v_{E}^{n} & \delta v_{N}^{n} & \delta v_{U}^{n} & \begin{array}{cccc} \begin{array}{ccc} \delta L & \delta \lambda & \delta h \end{array} & \varepsilon_{x}^{b} & \varepsilon_{y}^{b} & \varepsilon_{z}^{b} \end{array} & \nabla_{x}^{b} & \begin{array}{cc} \nabla_{y}^{b} & \nabla_{z}^{b} \end{array} \end{array}\right]}^{T}$$

The low-cost IMU in this work cannot measure the rotation rate of the Earth due to its insufficient accuracy. Moreover, the error caused by the change in g is minimal. Based on the chosen system state and the above-mentioned reasons, we can obtain the error-state equation through combining (4)–(9) [18]

(10) $$\dot{x}(t)=F(t)x(t)+G(t)w(t)$$

(11) $$F(t)={\left[\begin{array}{ccccc} -\omega_{in}^{n}\times_{3\times 3} & F_{12_{3\times 3}} & F_{13_{3\times 3}} & -C_{b_{3\times 3}}^{n} & 0_{3\times 3}\\ f^{n}\times_{3\times 3} & F_{22_{3\times 3}} & F_{23_{3\times 3}} & 0_{3\times 3} & C_{b_{3\times 3}}^{n}\\ 0_{3\times 3} & F_{32_{3\times 3}} & F_{33_{3\times 3}} & 0_{3\times 3} & 0_{3\times 3}\\ 0_{3\times 3} & 0_{3\times 3} & 0_{3\times 3} & 0_{3\times 3} & 0_{3\times 3}\\ 0_{3\times 3} & 0_{3\times 3} & 0_{3\times 3} & 0_{3\times 3} & 0_{3\times 3} \end{array}\right]}_{15\times 15}$$

(12) $$G(t)={\left[\begin{array}{ccccc} -C_{b_{3\times 3}}^{n} & 0_{3\times 3} & 0_{3\times 3} & 0_{3\times 3} & 0_{3\times 3}\\ 0_{3\times 3} & C_{b_{3\times 3}}^{n} & 0_{3\times 3} & 0_{3\times 3} & 0_{3\times 3}\\ 0_{3\times 3} & 0_{3\times 3} & 0_{3\times 3} & 0_{3\times 3} & 0_{3\times 3}\\ 0_{3\times 3} & 0_{3\times 3} & 0_{3\times 3} & 0_{3\times 3} & 0_{3\times 3}\\ 0_{3\times 3} & 0_{3\times 3} & 0_{3\times 3} & 0_{3\times 3} & 0_{3\times 3} \end{array}\right]}_{15\times 15}$$

(13) $$w(t)={\left[\begin{array}{lllllllll} n_{gx}^{b} & n_{gy}^{b} & n_{gz}^{b} & n_{ax}^{b} & n_{ax}^{b} & n_{ax}^{b} & \begin{array}{cccc} \begin{array}{ccc} 0 & 0 & 0 \end{array} & 0 & 0 & 0 \end{array} & 0 & \begin{array}{cc} 0 & 0 \end{array} \end{array}\right]}^{T}$$

where $-\omega_{in}^{n}\times$ and $f^{n}\times$ denote the 3 × 3 skew–symmetric matrix of $-\omega_{in}^{n}$ and $f^{n}$. $F_{12}$ (1,2) = $-\frac{1}{R_{M}+h}$, $F_{12}$ (2,1) = $\frac{1}{R_{N}+h}$, $F_{12}$ (3,1) = $\frac{\tan L}{\left(R_{N}+h\right)}$, $F_{13}$ (1,3) = $\frac{v_{N}^{n}}{{\left(R_{M}+h\right)}^{2}}$, $F_{13}$ (2,3) = $-\frac{v_{E}^{n}}{{\left(R_{N}+h\right)}^{2}}$, $F_{13}$ (3,1) = $\frac{v_{E}^{n}{\left(\sec L\right)}^{2}}{R_{N}+h}$, $F_{13}$ (3,3) = $-\frac{v_{E}^{n}\tan L}{{\left(R_{N}+h\right)}^{2}}$, $F_{22}$ (1,1) = $\frac{\tan L}{R_{N}+h}v_{N}^{n}-\frac{1}{R_{N}+h}v_{U}^{n}$, $F_{22}$ (1,2) = $\frac{\tan L}{R_{N}+h}v_{E}^{n}$, $F_{22}$ (1,3) = $-\frac{1}{R_{N}+h}v_{E}^{n}$, $F_{22}$ (2,1) = $-2\frac{\tan L}{R_{N}+h}v_{E}^{n}$, $F_{22}$ (2,2) = $-\frac{1}{R_{M}+h}v_{U}^{n}$, $F_{22}$ (2,3) = $-\frac{1}{R_{M}+h}v_{N}^{n}$, $F_{22}$ (3,1) = $2\frac{1}{R_{N}+h}v_{E}^{n}$, $F_{22}$ (3,2) = $2\frac{1}{R_{M}+h}v_{N}^{n}$, $F_{23}$ (1,1) = $\frac{{\left(\sec L\right)}^{2}}{R_{N}+h}v_{E}^{n}v_{N}^{n}$, $F_{23}$ (1,3) = $\frac{1}{{\left(R_{N}+h\right)}^{2}}v_{E}^{n}v_{U}^{n}-\frac{\tan L}{{\left(R_{N}+h\right)}^{2}}v_{E}^{n}v_{N}^{n}$, $F_{23}$ (2,1) = $-\frac{{\left(\sec L\right)}^{2}}{R_{N}+h}{\left(v_{E}^{n}\right)}^{2}$, $F_{23}$ (2,3) = $\frac{1}{{\left(R_{M}+h\right)}^{2}}v_{N}^{n}v_{U}^{n}+\frac{\tan L}{{\left(R_{N}+h\right)}^{2}}{\left(v_{E}^{n}\right)}^{2}$, $F_{23}$ (3,3) =$-\frac{1}{{\left(R_{M}+h\right)}^{2}}{\left(v_{N}^{n}\right)}^{2}-\frac{1}{{\left(R_{N}+h\right)}^{2}}{\left(v_{E}^{n}\right)}^{2}$, and the other elements in the $F_{12}$, $F_{13}$, $F_{22}$ and $F_{23}$ are zero. $F_{32}=M_{pv}$, $F_{33}=M_{pp}$. L represents the latitude. $n_{g}^{b}$ and $n_{a}^{b}$ represent the Gaussian white noise of the gyroscope and accelerometer in the *b*-frame.

### 2.3. Dual-Antenna Orientation Algorithm

The dual-antenna GNSS receiver defines the vehicle heading by calculating the baseline orientation. Thus, the primary aim of the algorithm is to form the baseline vectors by difference. For satellite *i* in Figure 2, the distance equations using the carrier wave can be expressed as [15]

(14) $$\lambda \varphi_{main}^{i}=r_{main}^{i}+c\left({\left(dt_{u}\right)}_{main}^{i}-{\left(dt_{s}\right)}_{main}^{i}\right)+\lambda N_{main}^{i}+{\left(d_{\mathrm{ion}}\right)}_{main}^{i}+{(d_{trop\;}}_{main}^{i}+{\left(\varepsilon_{mult}\right)}_{main}^{i}$$

(15) $$\lambda \varphi_{slave}^{i}=r_{slave}^{i}+c\left({\left(dt_{u}\right)}_{slave}^{i}-{\left(dt_{s}\right)}_{slave}^{i}\right)+\lambda N_{slave}^{i}+{\left(d_{\mathrm{ion}}\right)}_{slave}^{i}+{\left(d_{trop}\right)}_{slave}^{i}+{\left(\varepsilon_{mult}\right)}_{slave}^{i}$$

where λ represents the wavelength of the carrier, φ represents the phase difference, r represents the geometric distance between the satellite and the antenna, c is the speed of light, $dt_{u}$ and $dt_{s}$ represent receiver clock error and satellite clock error, N represents integer ambiguity, $d_{\mathrm{ion}}$ and $d_{\mathrm{trop}}$ represent ionospheric and tropospheric errors, and $\varepsilon_{mult}$ represents multipath error.

Then, through two differential operations, the double difference equation can be obtained as

(16) $$\lambda \Delta \nabla \varphi_{main,slave}^{ij}=\Delta \nabla r_{main,slave}^{ij}+\lambda \Delta \nabla N_{main,slave}^{ij}$$

A transformation of the equation gives

(17) $$\lambda \Delta \nabla \varphi_{main,slave}^{ij}=(l_{r}^{i}-l_{r}^{j})\cdot b_{main,slave}+\lambda \Delta \nabla N_{main,slave}^{ij}$$

where $l_{r}$ represents the identity direction vector between the antenna and the satellite, $b_{main,slave}$ represents the baseline vector, and Δ∇ represents the double difference operator.

The Least-squares Ambiguity Decorrelation Adjustment (LAMBDA) algorithm is utilized to solve for integer ambiguity [31]. The least squares method is used to obtain the baseline vector.

### 2.4. Measurement Model

#### 2.4.1. Initial Measurement Model

In order to develop the measurement model, the heading angle, velocity, and position from the GNSS receiver are imported into the measurement error equation. The measurement model can be expressed as

(18) $$\left[\begin{array}{c} \psi_{I}-\psi_{G}\\ v_{I}^{n}-v_{G}^{n}\\ p_{I}^{n}-p_{G}^{n} \end{array}\right]=\left[\begin{array}{ccc} 1_{1\times 1} & 0_{1\times 1} & 0_{1\times 1}\\ 0_{3\times 3} & I_{3\times 3} & 0_{3\times 3}\\ 0_{3\times 3} & 0_{3\times 3} & I_{3\times 3} \end{array}\right]\left[\begin{array}{c} \delta \psi_{I}\\ \delta v_{I}^{n}\\ \delta p_{I}^{n} \end{array}\right]+\left[\begin{array}{c} \delta \psi_{G}\\ \delta v_{G}^{n}\\ \delta p_{G}^{n} \end{array}\right]$$

where $\psi_{G}$, $v_{G}^{n}$ and $p_{G}^{n}$ are measured by GNSS, and $\psi_{I}$, $v_{I}^{n}$ and $p_{I}^{n}$ are measured by INS.

#### 2.4.2. The Lever Arm Correction Algorithm

Since the origin of the measured position and velocity by GNSS is located at the phase center of the main antenna and the origin of the INS measurements is located at the IMU, the spatial distance between the main antenna and the IMU (referred to as the lever arm) should be taken into account.

The IMU is located at the b-frame origin; GNSS velocity can be corrected by [32]

(19) $$v_{G}^{n}=v_{G}^{n}\prime -C_{b}^{n}\left(\omega_{eb}^{b}\times \delta l^{b}\right)$$

where $v_{G}^{n}$ is the velocity at the origin of the *n*-frame, $v_{G}^{n}\prime$ is the velocity prior to correction, $\omega_{eb}^{b}$ is approximately equal to $\omega_{ib}^{b}$ because $\omega_{eb}^{b}=\omega_{ib}^{b}-\omega_{ie}^{b}$ and the value of $\omega_{ie}^{b}$ is minimal, $\delta l^{b}$ is the spatial distance of the main antenna from the IMU, and $\delta l^{b}={\left[\begin{array}{ccc} 0 & -\frac{B}{2} & 0 \end{array}\right]}^{T}$ (the placement can be seen in Figure 2).

The GNSS position can be corrected by [32]

(20) $$p_{G}^{n}=p_{G}^{n}\prime -M_{pv}C_{b}^{n}\delta l^{b}$$

where $p_{G}^{n}$ is the position at the origin of the n-frame, and $p_{G}^{n}\prime$ is the position prior to correction.

#### 2.4.3. Corrected Measurement Model Using the Kinematic Constraint

Considering the actual conditions of the vehicle in motion, the vehicle is moving normally without skidding and jumping. The kinematic constraint, which refers to zero velocity in the lateral and vertical directions of vehicle movement, is utilized to improve the accuracy of the measurement model:

(21) $$v_{G}^{n}=\left[\begin{array}{c} v_{x}^{n}\\ v_{y}^{n}\\ v_{z}^{n} \end{array}\right]=\left[\begin{array}{c} 0\\ v_{y}^{n}\\ 0 \end{array}\right]$$

Using the corrected GNSS data, the measurement equation can eventually be expressed as

(22) Z(t)=H(t)x(t)+υ(t)

(23) $$Z(t)={{\left[\begin{array}{ccc} {\left(\psi_{I}-\psi_{G}\right)}_{1\times 1} & {\left(v_{I}^{n}-v_{G}^{n}\right)}_{3\times 1} & {\left(p_{I}^{n}-p_{G}^{n}\right)}_{3\times 1} \end{array}\right]}^{T}}_{7\times 1}$$

(24) $$H(t)={\left[\begin{array}{ccccc} H_{11_{3\times 3}} & 0_{3\times 3} & 0_{3\times 3} & 0_{3\times 3} & 0_{3\times 3}\\ 0_{3\times 3} & I_{3\times 3} & 0_{3\times 3} & 0_{3\times 3} & 0_{3\times 3}\\ 0_{3\times 3} & 0_{3\times 3} & I_{3\times 3} & 0_{3\times 3} & 0_{3\times 3}\\ 0_{3\times 3} & 0_{3\times 3} & 0_{3\times 3} & 0_{3\times 3} & 0_{3\times 3}\\ 0_{3\times 3} & 0_{3\times 3} & 0_{3\times 3} & 0_{3\times 3} & 0_{3\times 3} \end{array}\right]}_{15\times 15}$$

where $H_{11}$ (3,3) = 1, and the other elements in the $H_{11}$ are zero.

(25) $$\upsilon (t)={\left[\begin{array}{ccc} \delta \psi_{G} & \delta v_{G}^{n} & \delta p_{G}^{n} \end{array}\right]}^{T}$$

where $\delta \psi_{G}$, $\delta v_{G}^{n}$, and $\delta p_{G}^{n}$ are the measurement noises, which correspond to the one-dimensional heading error, three-dimensional velocity error vector, and three-dimensional position error vector of the GNSS receiver, respectively.

#### 2.4.4. Corrected Measurement Model for Special Scenarios

When the GNSS receiver can fix positions but the dual-antenna orientation algorithm fails, the measurement model can be changed to

(26) $$Z(t)={{\left[\begin{array}{cc} {\left(v_{I}^{n}-v_{G}^{n}\right)}_{3\times 1} & {\left(p_{I}^{n}-p_{G}^{n}\right)}_{3\times 1} \end{array}\right]}^{T}}_{6\times 1}$$

(27) $$H(t)={\left[\begin{array}{ccccc} 0_{3\times 3} & 0_{3\times 3} & 0_{3\times 3} & 0_{3\times 3} & 0_{3\times 3}\\ 0_{3\times 3} & I_{3\times 3} & 0_{3\times 3} & 0_{3\times 3} & 0_{3\times 3}\\ 0_{3\times 3} & 0_{3\times 3} & I_{3\times 3} & 0_{3\times 3} & 0_{3\times 3}\\ 0_{3\times 3} & 0_{3\times 3} & 0_{3\times 3} & 0_{3\times 3} & 0_{3\times 3}\\ 0_{3\times 3} & 0_{3\times 3} & 0_{3\times 3} & 0_{3\times 3} & 0_{3\times 3} \end{array}\right]}_{15\times 15}$$

When the GNSS does not receive the signal and cannot fix positions, none of the GNSS receiver data are available, so the barometer and INS data are used to ensure short-time accuracy.

The barometer/INS measurement model can be expressed as

(28) Z(t)=H(t)x(t)+υ(t)

(29) $$Z(t)={{\left[h_{I}^{n}-h_{B}^{n}\right]}^{T}}_{1\times 1}$$

where $H_{15\times 15}$ (6,6) = 1, and the other elements in the $H_{15\times 15}$ are zero. υ(t) indicates barometer measurement noise.

### 2.5. AKF Design

Based on the models developed in Section 2.2 and Section 2.4, an AKF is designed for sensor data fusion. The discretization of a linearized system in continuous time leads to [33]

(30) $$\{\begin{array}{c} x\left(k\right)=\Phi_{k/k-1}x\left(k-1\right)+G_{k/k-1}W\left(k-1\right)\\ Z\left(k\right)=H\left(k\right)x\left(k\right)+V\left(k\right) \end{array}$$

where $\Phi_{k/k-1}\approx I+F(t_{k-1})T_{s}$, $T_{s}$ is the discrete interval time.

The state equation corresponds to Equations (10)–(12). The measurement equation contains three cases, corresponding to Equations (22)–(24), (26), (27), and (28), (29), respectively.

AKF update is mainly divided into two parts: prediction and correction. The prediction part is divided into one-step state prediction and MSE (mean square error) matrix prediction. The formulas are as follows [34]:

(31) $${\hat{x}}_{k/k-1}=\Phi_{k/k-1}{\hat{x}}_{k-1}$$

(32) $$P_{k/k-1}=\Phi_{k/k-1}P_{k-1}\Phi_{k/k-1}^{T}+G_{k-1}Q_{k-1}G_{k-1}^{T}$$

The prediction residual is defined by

(33) $${\hat{V}}_{k}=Z_{k}-H_{k}{\hat{x}}_{k/k-1}$$

The prediction residual is constructed by a two-segment adaptive factor [35]:

(34) $$\alpha_{k}=\{\begin{array}{c} 1\begin{array}{ccc} & \left|{\tilde{V}}_{k}\right|\leq c & \end{array}\\ \frac{c}{\left|{\tilde{V}}_{k}\right|}\begin{array}{ccc} & \left|{\tilde{V}}_{k}\right|>c & \end{array} \end{array}$$

where $\alpha_{k}$ is the adaptive factor, ${\tilde{V}}_{k}=∥{\hat{V}}_{k}∥/\sqrt{trace\left(P_{{\hat{V}}_{k}}\right)}$, $P_{{\hat{V}}_{k}}$ is the prediction residual covariance matrix, $P_{{\hat{V}}_{k}}=H_{k}P_{k,k-1}H_{k}+R_{k}$. ${∥}^{*}∥$, and $trace{(}^{*})$ represents the least-squares norm and the trace of the matrix, respectively. c is the empirical threshold.

In the correction part, Kalman gain is calculated first, followed by state estimation and MSE matrix estimation. The formulas are as follows.

(35) $$K_{k}=\frac{1}{\alpha_{k}}P_{k/k-1}H_{k}^{T}{\left(\frac{1}{\alpha_{k}}H_{k}P_{k/k-1}H_{k}^{T}+R\right)}^{-1}$$

(36) $${\hat{x}}_{k}={\hat{x}}_{k/k-1}+K_{k}\left(Z_{k}-H_{k}{\hat{x}}_{k/k-1}\right)$$

(37) $$P_{k}=\frac{1}{\alpha_{k}}\left(I-K_{k}H_{k}\right)P_{k/k-1}$$

## 3. Experiment and Discussion

### 3.1. Experiment Platform

**Figure 3:**
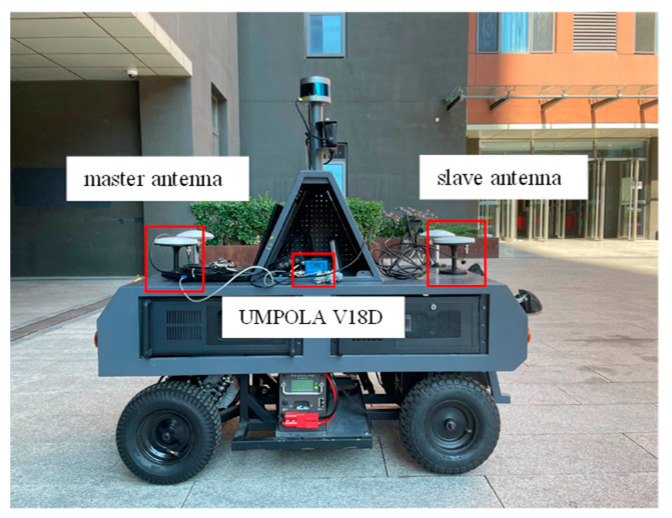
The experiment vehicle.

Figure 3 shows the experimental vehicle platform equipped with an integrated navigation receiver (UMPOLA V18D, UniStrong, Beijing, China) consisting of a GNSS receiver, an IMU module, and a barometer. The characteristics of the three modules are listed in Table 1. In order to obtain data from the different modules at the same time, all information is collected using NAVITEST engineering software version 1.0.0.1 and stored on the PC.

The positioning antenna (master antenna) is located at the rear limit hole of the vehicle and the directional antenna (slave antenna) is located at the front limit hole. The two antennas are 1.0 m apart.

### 3.2. Heading Angle Experiment and Discussion

#### 3.2.1. Open and Occluded Environment Experiment

**Figure 4:**
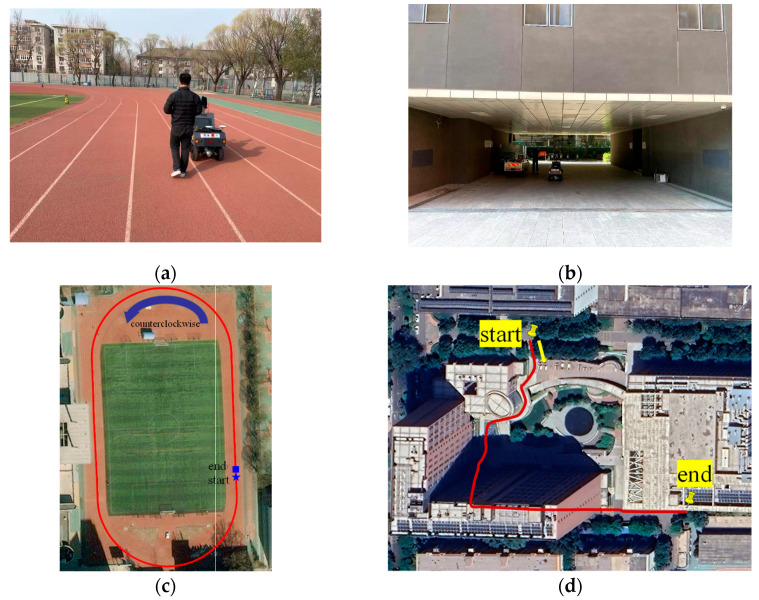
Acquisition of experimental data in the open and occluded environment: (**a**) Experiment with real-time scenario in the playground; (**b**) Test vehicle through the building tunnel; (**c**) Test vehicle trajectory in the open environment; (**d**) Test vehicle trajectory in the occluded environment.

The playground of Beijing Forestry University has both straight and curved paths and is not obstructed by tall buildings and trees, so it can intuitively and clearly reflect the performance of the proposed algorithm. The open environment experimental data were obtained by the vehicle traveling one lap counterclockwise around the fifth track of the playground. Considering the inevitability of the occlusion problem, the occluded environment experiments at the east campus were added to the heading angle estimation experiments. The trajectory of the occluded environment experiment included sharp curves, a ninety-degree turn, and a straight line, and these roads verified the sensitivity of the algorithm. The experimental scenario and trajectory of the open and occluded environment are depicted in Figure 4.

**Figure 5:**
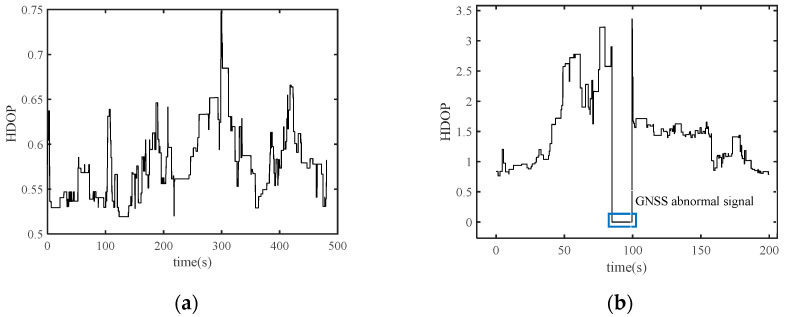
HDOP during the open and occluded environment experiment: (**a**) The value of HDOP during the open environment experiment; (**b**) The value of HDOP during the occluded environment experiment.

In order to clearly characterize the degree of obscuration, horizontal dilution of precision (HDOP) was introduced into the experiment. As shown in Figure 5a, the HDOP values were below 1 throughout the experiment, which means that the GNSS signal was good. The value of HDOP exhibits significant fluctuations in Figure 5b. At 85–100 s, the vehicle drove into the building tunnel (Figure 4b); at this time, the GNSS signal was completely obstructed and the GNSS receiver did not work properly.

**Figure 6:**
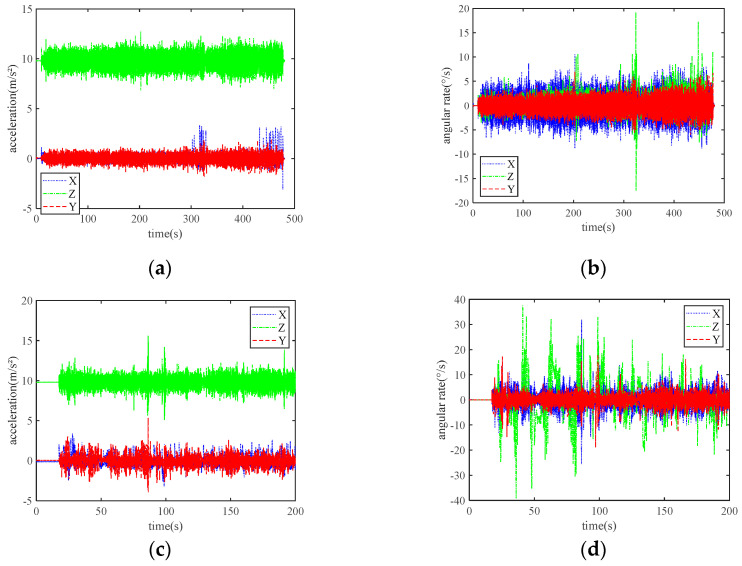
Acquisition of original IMU data in the open and occluded environments: (**a**) Original triaxial accelerometer data in the playground; (**b**) Original triaxial gyroscope data in the playground; (**c**) Original triaxial accelerometer data in the occluded environment; (**d**) Original triaxial gyroscope data in the occluded environment.

As shown in Figure 6a,b, the maximum lateral acceleration and angular rate reached 3.33 m/s^2^ and 19.21°/s in the open environment experiment. The acceleration and angular rate produced even more dramatic fluctuations, as shown in Figure 6c,d, where the peak of the angular rate exceeded 35°/s. This result is highly compatible with the route of the occluded environment experiment.

#### 3.2.2. Experimental Results and Discussion

**Figure 7:**
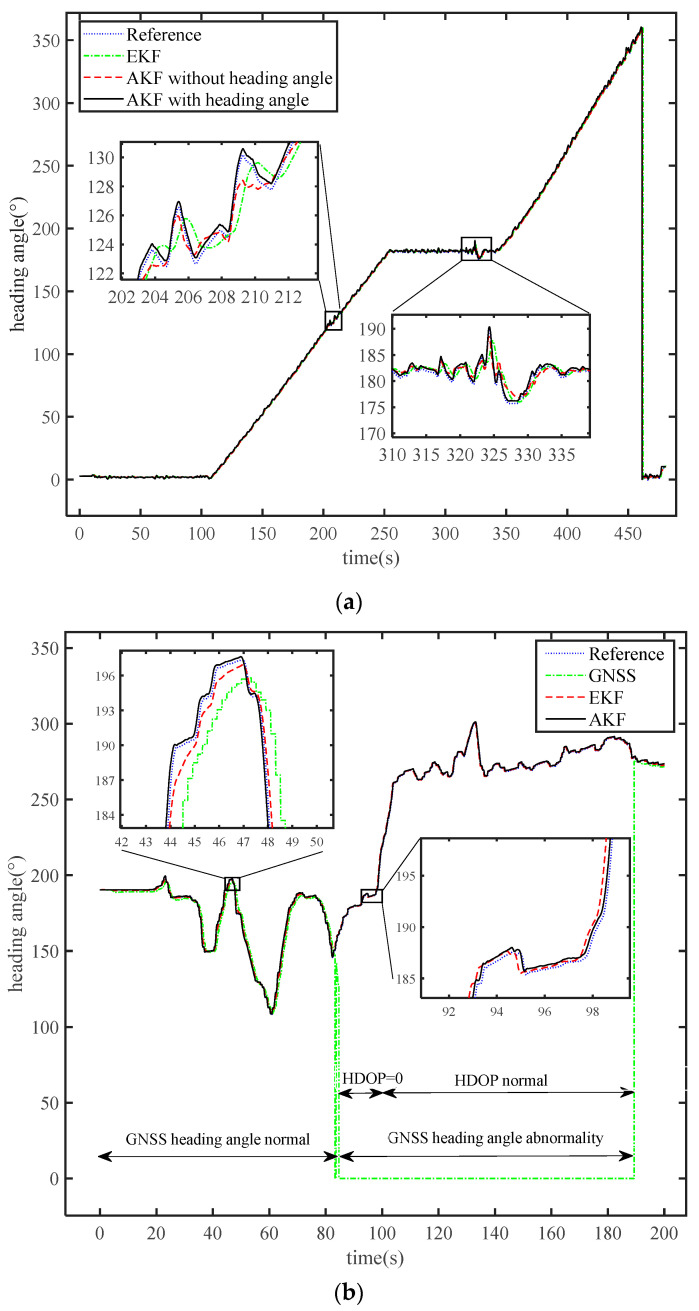
The estimation of heading angle during the open and occluded environment experiments: (**a**) The estimation of heading angle in the open environment; (**b**) The estimation of heading angle in the occluded environment.

The heading angle estimations for the open and occluded environment experiments are illustrated in Figure 7. The reference data are the result of using the original filtering algorithm of the integrated navigation receiver. The accuracy of the reference heading angle data is 0.05° at the one-meter baseline, which is better than the accuracy of the GNSS orientation algorithm.

In Figure 7a, the start and end heading angle of the test vehicle varied by more than 360°. The extended Kalman filter (EKF) method was selected as the comparison method and the measurement variables of the EKF method were three-dimensional velocity and position. As can be seen from the two local magnifications, the curves of the AKF method are more identical to the reference curve than that of the EKF method, regardless of whether the GNSS heading angle is utilized as a measurement variable or not. When heading angle is used as a measurement variable, the results of the AKF with heading angle method are significantly closer to the reference value than those of the AKF without heading angle method.

The results of the heading angle comparison using the different methods are presented in Figure 7b. The results of the 200-s experiment were divided into three parts: 0–85 s for part 1, 85–189 s for part 2, and 189–200 s for part 3. In part 2, the time period of the GNSS heading angle anomaly diverges from the results of HDOP in Figure 5b. HDOP was zero from 85 s to 100 s, which means that the GNSS receiver could not fix the position. At this point, the only measurement variable was the elevation data from the barometer. The results of the proposed method maintain better accuracy than the comparison method. At the moment of the 100th second, the value of HDOP changed abruptly from 0 to 3.36 and the positioning function was restored. The GNSS heading angle did not return to normal until the 189th second, as the dual-antenna orientation algorithm failed. The measurement variables of the AKF method are three-dimensional velocity and position. Due to the GNSS heading angle abnormality in the whole of part 2, the measurement variables of the proposed method did not include the GNSS heading angle. Successful experimental results using the AKF method were presented in situations with both complete and partial occlusion. As shown in the two enlargements, after integration with the high-frequency INS, the estimated heading by the AKF method was smoother than that of the original GNSS heading. The AKF curve has less error compared to the curve of the EKF method.

In order to analyze the results accurately, Table 2 exhibits specific error indicators throughout the experimental period. The values of MAE, STD, and RMS represent the overall level of error, which mainly depends on the environment and the algorithm. By comparing different scenarios, it is revealed that the errors in the open environment are overall better than the errors in the occluded environment. In both scenarios, the minimum error values were obtained by the AKF method, which means that the proposed method has the best accuracy. The value of MAX appeared at a certain moment and showed the instantaneous error of the applied methodology. In the open scenario, the maximum errors of the EKF and AKF without heading angle methods occurred at 325.2 and 326.9 s, respectively. As depicted in Figure 7a, these two moments are just after the peak, which was formed by the sudden lateral acceleration from the operator. Therefore, the value of MAX can also be affected by human manipulation in addition to the environment and algorithm.

For the errors in the open environment, the four indicators of the heading angle error of the two AKF methods are all better than the error value of the EKF method. MAE, MAX, and STD decreased by 30.49%, 81.99%, and 61.60%, respectively, compared to the error of the EKF method. In AKF with heading angle, RMS, which is the most commonly used index for evaluating navigation accuracy, was also 37.62% less than the value of RMS in the EKF method. This reduction proves that the algorithm of the AKF with heading angle method improved the heading angle accuracies in the open environment.

In the occluded environment, a significant increase in the GNSS heading angle error occurred due to severe obstruction of the GNSS signals. But the errors of heading obtained by the AKF method maintained a high level of accuracy. Compared to the error values of the EKF method, the four error values of the AKF method are 0.5048° (MAE), 2.9052° (MAX), 0.5098° (STD), and 0.6367° (RMS) in turn, which represent 35.24%, 58.37%, 57.07%, and 47.37%. The error fluctuations caused by environment occlusion were effectively suppressed by the AKF method.

Based on the aforementioned discussion, the accuracy of the heading angle was effectively improved by the AKF algorithm proposed in this study. Additionally, the GNSS/INS/Barometer integration using AKF with heading angle has better stability and robustness than the EKF method.

The experimental results also demonstrate that the open environment without the occlusion of buildings and trees is the primary condition for the proposed algorithm to reduce the error. Although the sensitivity of the proposed algorithm is better than the comparative methods, a slight hysteresis still exists. As shown in Figure 7, the reaction rate of the proposed algorithm is still slightly slower than the reference results when the vehicle’s heading is rapidly changed by the operator. If the test vehicle is required to travel in a straight line, reducing the lateral acceleration contributes to decreasing the sharp increase in errors.

## 4. Conclusions

A dual-antenna GNSS receiver was added to this study to improve the vehicle heading angle accuracy. A RANSAC method was proposed to improve the initial heading angle accuracy. By using the dual-antenna orientation algorithm, GNSS heading angle was added as a measurement variable in the integrated navigation system based on AKF in addition to three-dimensional position and velocity. Moreover, velocity and position were corrected by the lever arm correction algorithm. The kinematic constraint, which refers to zero velocity in the lateral and vertical directions of vehicle movement, was utilized to enhance the accuracy of the measurement model. When none of the GNSS receiver data were available, a barometer was added to the traditional GNSS/INS integrated navigation system to enhance the reliability of the system. INS data and the barometer instead of GNSS were used to ensure short-time accuracy. Through the fusion of the INS error equation with the measurement equation, a dual-antenna GNSS/Barometer/INS integration based on AKF was created.

The proposed dual-antenna GNSS/INS/Barometer integration using the AKF algorithm was validated in open and occluded environments. In the open environment, the heading angle accuracy of the AKF with heading angle method was 0.4435° (MAE), 1.1489° (MAX), 0.3264° (STD), and 0.5418° (RMS), which represent reductions of 30.49%, 81.99%, 61.60%, and 37.62% compared to the EKF method, respectively. In the occluded environment, the AKF algorithm maintained a high level of heading accuracy. The heading angle accuracy of the proposed method was 0.636° (RMS), which is 47.37% less than the error of the EKF method. The experimental results indicate that the proposed dual-antenna GNSS/Barometer/INS integration based on the AKF method effectively improves the vehicle heading angle accuracy, and the error values are also superior to those of the EKF method in both open and occluded environments.

Due to time constraints, the number of comparison methods was limited. Therefore, future work should be dedicated to researching new data fusion methods. Improving sensitivity is one of the targets of the algorithms when the vehicle heading changes rapidly. Non-linear filters should also be a focus of study.

## Figures and Tables

**Table 1:** Characteristics of GNSS, IMU, and barometer module.

Module		Specifications	Performance
GNSS		Position accuracy (RMS *)	1.2 m (Plane), 2.5 m (Elevation)
		Time accuracy	10 ns
		Velocity accuracy (RMS)	0.02 m/s
		Measurement frequency	5 Hz
IMU	Gyroscope	Range	±400°/s
		Bias stability	10°/h
		Bias Repeatability	10°/h
		Measurement frequency	100 Hz
	Accelerometer	Range	±5 g
		Bias stability	0.2 mg
		Bias Repeatability	0.2 mg
		Measurement frequency	100 Hz
Barometer		Height accuracy	10 cm
		Measurement frequency	5 Hz

* RMS is Root Mean Square.

**Table 2:** Heading angle errors during the open and occluded environment experiment.

Scenario	Method	MAE (°) ^1^	MAX (°) ^2^	STD (°) ^3^	RMS (°)
the open environment	EKF	0.6380	6.3795	0.8500	0.8686
	AKF without heading angle	0.4857	2.6924	0.5956	0.6174
	AKF with heading angle	0.4435	1.1489	0.3264	0.5418
the occluded environment	GNSS ^4^	2.6789	16.3810	3.6731	3.7129
	EKF	0.7795	6.9785	1.1874	1.2090
	AKF	0.5048	2.9052	0.5098	0.6367

^1^ MAE is Mean Absolute Error. ^2^ MAX is Maximum. ^3^ STD is Standard Deviation. ^4^ GNSS data range from 1 to 82 s.

## Data Availability

Data are contained within the article.
